# Multilevel competing risk models to evaluate the risk of nosocomial infection

**DOI:** 10.1186/cc13821

**Published:** 2014-04-08

**Authors:** Martin Wolkewitz, Ben S Cooper, Mercedes Palomar-Martinez, Francisco Alvarez-Lerma, Pedro Olaechea-Astigarraga, Adrian G Barnett, Stephan Harbarth, Martin Schumacher

**Affiliations:** 1 Institute of Medical Biometry and Medical Informatics, University Medical Center Freiburg, Freiburg, Germany; 2 Freiburg Center of Data Analysis and Modelling, Albert-Ludwigs University Freiburg, Freiburg, Germany; 3 Mahidol University, Bangkok, Thailand; 4 Hospital Universitari Arnau de Vilanova, Lleida, Universitat Autónoma de Barcelona, Barcelona, Spain; 5 Service of Intensive Care Medicine, Parc de Salut Mar, Barcelona, Spain; 6 Service of Intensive Care Medicine, Hospital de Galdakao-Usansolo, Bizkaia, Spain; 7 Institute of Health and Biomedical Innovation and School of Public Health and Social Work, Queensland University of Technology, Brisbane, QLD 4059, Australia; 8 Geneva University Hospitals, Geneva, Switzerland

## Abstract

**Introduction:**

Risk factor analyses for nosocomial infections (NIs) are complex. First, due to competing events for NI, the association between risk factors of NI as measured using hazard rates may not coincide with the association using cumulative probability (risk). Second, patients from the same intensive care unit (ICU) who share the same environmental exposure are likely to be more similar with regard to risk factors predisposing to a NI than patients from different ICUs. We aimed to develop an analytical approach to account for both features and to use it to evaluate associations between patient- and ICU-level characteristics with both rates of NI and competing risks and with the cumulative probability of infection.

**Methods:**

We considered a multicenter database of 159 intensive care units containing 109,216 admissions (813,739 admission-days) from the Spanish HELICS-ENVIN ICU network. We analyzed the data using two models: an etiologic model (rate based) and a predictive model (risk based). In both models, random effects (shared frailties) were introduced to assess heterogeneity. Death and discharge without NI are treated as competing events for NI.

**Results:**

There was a large heterogeneity across ICUs in NI hazard rates, which remained after accounting for multilevel risk factors, meaning that there are remaining unobserved ICU-specific factors that influence NI occurrence. Heterogeneity across ICUs in terms of cumulative probability of NI was even more pronounced. Several risk factors had markedly different associations in the rate-based and risk-based models. For some, the associations differed in magnitude. For example, high Acute Physiology and Chronic Health Evaluation II (APACHE II) scores were associated with modest increases in the rate of nosocomial bacteremia, but large increases in the risk. Others differed in sign, for example respiratory vs cardiovascular diagnostic categories were associated with a reduced rate of nosocomial bacteremia, but an increased risk.

**Conclusions:**

A combination of competing risks and multilevel models is required to understand direct and indirect risk factors for NI and distinguish patient-level from ICU-level factors.

## Introduction

Nosocomial infections (NIs) are a major threat for hospitalized patients, particularly in intensive care units (ICUs), because they are associated with increased mortality and morbidity [1,2]. Analysis of data from large multicenter studies has the potential to improve our understanding of how patient- and ICU-level characteristics impact NI outcomes. Such analysis is, however, complicated by two factors: unexplained ICU-level variation and the importance of competing risks.

First, there are endogenous and exogenous modes of NI acquisition [3]. Harbarth *et al*. [3] showed that about 20% of NIs are exogenous and therefore potentially preventable. Potential transmission routes of exogenous NIs are contact with contaminated environmental surfaces or cross-transmission via health-care workers or patients. Thus, patients from the same ICU who share the same environmental exposure are likely to be more similar with regard to acquiring a NI than patients from different ICUs. In addition to patient-individual characteristics, ICU-specific factors, such as number of beds and nurses, type of ICU and infection control policies, are potential determinants for the occurrence of NI. To distinguish patient-level and ICU-specific factors requires multilevel analysis, but this is rarely used in hospital epidemiology [4]. The clustered structure of the data (patients within ICUs) often contains information that can be of value in understanding associations between risks and NIs [5].

Second, the length of ICU stay is a key determinant of the risk of NI. However, most patients are discharged from ICU or die in ICU without NI. Factors that are associated with a high increased rate of infection are often also associated with an increased risk of dying in the hospital as well as with an extended length of stay. These competing events play an important role in risk interpretation of NI and make extended survival models necessary [6-9]. Again, rates of NI, discharge and death without NI might also depend on patient- as well as on ICU-level factors. Thus, a combination of extended survival and multilevel models is required to understand how different risk factors impact NI outcomes. The aim of this paper is to apply established and innovative statistical methods [10,11] to investigate heterogeneity in risks and rates across ICUs of NI and concurrently occurring competing events.

## Material and methods

### Spanish ICU data

We used a multicenter database from the Spanish surveillance network HELICS-ENVIN [12], embedded in the HELICS project (Hospitals in Europe Link for Infection Control through Surveillance). The reliability and quality of the surveillance program has recently been investigated [13]. Data were prospectively collected on an individual patient level and also aggregate ICU level. For our purpose, we included ICUs that contributed to the registry between January 2006 and December 2011 and we included only patients who stayed at least two days in ICU. We excluded ICUs that contributed less than 100 patient admissions to the cohort to reduce artificial heterogeneity. To get a robust outcome, we focused on primary or secondary nosocomial bacteremia (NB). The study population, 159 intensive care units with 109,216 admissions (813,739 admission-days), is summarized in Table 1. The data of this official surveillance are encrypted and completely anonymous. Patients’ consent was not needed. This Deutsche Forschungsgemeinschaft research project was approved by the ethics committee of the University Medical Center Freiburg, Germany.

**Table 1:** Description of study population

**General**	**Frequency**	
Number of admissions	109,216	
Number of admission-days	813,739	
Number of ICUs	159	
Number of nosocomial bacteremia during ICU stay	5,498 (5.03%)	
Number of deaths without NB during ICU stay	12,678 (11.61%)	
Number of discharges without NB from ICU	90,142 (82.54%)	
Number of administrative censored admissions	898 (0.82%)	
Overall risk of nosocomial bacteremia (censored excluded)	5.08%	
Overall rate of nosocomial bacteremia	6.75 / 1,000 admission-days	

**Risk factors**	**Frequency (%)**	**Frequency (%)**
	**(patient level)**	**(ICU level)**
**ICU / hospital level covariates**		
Number of beds in ICU:		
0 to 10 (reference)	30,389 (27.82)	61 (38.36)
11 to 20	46,524 (42.60)	71 (44.65)
21 to 30	21,668 (19.84)	19 (11.95)
31 to 40	7,673 (7.03)	5 (3.14)
> 40	2,962 (2.71)	3 (1.89)
Number of beds in hospital:		
0 to 500 (reference)	52,426 (48.00)	94 (59.12)
501 to 1,000	8,259 (7.56)	10 (6.29)
> 1,000	48,531 (44.44)	55 (34.59)
Type of hospital:		
Private	6,541 (5.99)	12 (7.55)
Public	102,675 (94.01)	147 (92.45)
Type of ICU:		
Polyvalent (reference)	96,478 (88.34)	138 (86.79)
Medical	3,895 (3.57)	5 (3.14)
Surgery	3,103 (2.84)	3 (1.89)
Coronary	525 (0.48)	2 (1.26)
Traumatology	3,273 (3.00)	6 (3.77)
Post-surgery cardiology	1,834 (1.68)	4 (2.52)
Burn	108 (0.10)	1 (0.63)
University+teaching hospital (reference)	67,917 (62.19)	86 (54.09)
Teaching hospital (no university)	30,089 (27.55)	52 (32.70)
Hospital without teaching/university	11,210 (10.26)	21 (13.21)
**Calendar year of admission**		
2006 (reference)	14,318 (13.11)	
2007	17,819 (16.32)	
2008	21,559 (19.74)	
2009	25,660 (23.49)	
2010+	29,860 (27.34)	
**Patient level covariates**		
APACHE II score:		
0 to 10 (reference)	40,353 (36.95)	
11 to 20	44,654 (40.89)	
21 to 30	19,191 (17.57)	
> 30	5,018 (4.59)	
Age (years):		
0 to 40	54,477 (49.88)	
41 to 60	12,931 (11.84)	
61 to 80 (reference)	29,371 (26.89)	
> 80	12,437 (11.39)	
Days in hospital before ICU admission:		
0 to 3 (reference)	87,208 (79.85)	
4 to 6	5,864 (5.37)	
7 to 10	4,962 (4.54)	
> 10	11,182 (10.24)	
Type of diagnosis:		
Cardiovascular (reference)	54,374 (49.79)	
Respiratory	15,243 (13.96)	
Gastrointestinal	14,626 (13.39)	
Central nervous system	17,567 (16.08)	
Other diagnoses	7,406 (6.78)	
Antibiotic treatment 48 h before and/or after ICU admission	23,178 (21.22)	
Gender (male)	71,223 (65.21)	
Origin: community (reference)	54,996 (50.36)	
Origin: hospital/ICU	54,220 (49.64)	
Trauma	8,927 (8.17)	

### Shared frailty models for competing risks

#### Model 1: etiologic model (rate-based)

The classic way to analyze competing risks data is to study event-specific hazard rates, i.e., fitting a proportional hazard model for each event (NB, death without NB and discharge without NB) separately. Random effects for each ICU (i.e. frailties) can be introduced by a shared gamma frailty model [10,14] (see details in Additional file 1). For each of the three events (NB, death and discharge), we fitted models to assess heterogeneity for NB rates, quantified by the corresponding variance estimator *θ*. Large variances signify a closer similarity between patients within ICU and greater heterogeneity across ICUs. The following quantities were calculated for each of the three events: the baseline hazard, ICU effects, variance of ICU effects (*θ*) and the hazard ratios for multilevel risk factors at the patient and ICU levels. In this approach, the hazards, i.e., the *daily* risks of the primary outcome (NB) and the competing events (death or discharge without NB), are studied. Note that the hazard of NB does not depend on the competing events.

#### Model 2: predictive model (risk-based)

The cumulative incidence function of NB is defined as the probability of NB over a period of time and interpreted as the actual risk of NB occurring in this time period. This approach is useful for predicting NB. It has previously been shown that the way in which risk factors are associated with the NB hazard (instantaneous risk) may not coincide with the way these factors are associated with the cumulative incidence of NB (cumulative risk) [15]. To study the risk (cumulative incidence function) of NB, we used the Fine and Gray model [16] and introduced a shared frailty structure to investigate heterogeneity in a similar way as Katsahian *et al*. [11]. Using this model we calculated the following quantities: the baseline subdistribution hazard and corresponding cumulative incidence of NB, ICU effects, variance of ICU effects (*θ*) and the subdistribution hazard ratios for multilevel risk factors at the patient and ICU levels. In this approach, the *cumulative* risk of the primary outcome (NB) is studied. In contrast to the event-specific approach, the cumulative risk of NB depends on the NB hazard as well as on the hazards of the competing events and it tends towards the overall risk, i.e., the incidence proportion of NB.

For both models, we first used a model with frailties for each ICU but without covariates (null model). Then, we considered a multivariate model by introducing patient-individual as well as ICU-specific covariates and estimated the frailties for each ICU. For all analyses we used the flexible R package *frailtyPack*[17].

## Results

In the following we present the detailed results for primary and secondary nosocomial bacteremia (NB).

### Baseline hazard rates and cumulative incidence function

**Figure 1:**
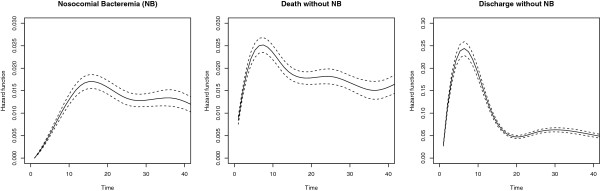
**Estimated baseline hazard functions for the three outcomes.** Data are from the null model without covariates. Associated 95% confidence intervals are shown as broken lines.

**Figure 2:**
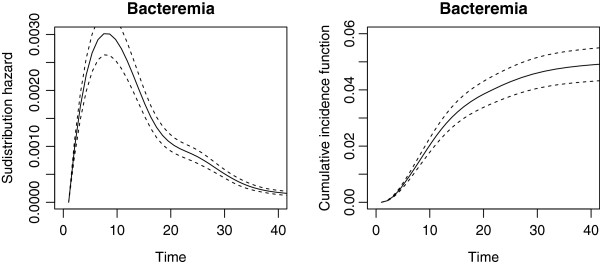
**Estimated subdistribution hazard function (left) and cumulative incidence function (right).** Data are from the shared frailty model for the subdistribution hazard of NB without covariates. The subdistribution hazard is shown as the black curves and associated 95% confidence intervals as broken lines.

The overall baseline hazard rates based on the null models without covariates are shown for each event in Figure 1. The hazard rate of NB is increasing with the time from admission and has a peak at day 15; for instance, the daily risk of acquiring a NB at day 10 (day 30) for a patient is about 1% (1.5%) given that he or she has stayed at ICU without a NB for at least 9 days (29 days). The death hazard rate without NB is about 2% for the first 40 days from admission. Obviously, the discharge hazard rate without NB is the strongest hazard with its peak (about 25%) about 5 to 6 days after admission and a strong decrease afterwards; meaning the likelihood of discharge without NB decreases for each survived day in ICU. The subdistribution hazard function and cumulative incidence function of NB are displayed in Figure 2; the cumulative incidence function tends towards the overall risk of 5.08%.

### Heterogeneity across ICUs

**Figure 3:**
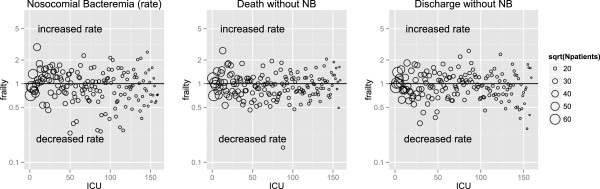
**Estimated frailties (random effects) for each ICU for the three outcomes.** Data are from the null model without covariates. The ICUs are ordered on the *X*-axis according to the contributing number of patients; the circles are proportional to the magnitude of contribution of the ICU, e.g., an ICU with sqrt(Npatients) = 60 contributed 60^2^ = 3600 admissions to the cohort. The *Y*-axis has a log scale. NB, nosocomial bacteremia.

**Figure 4:**
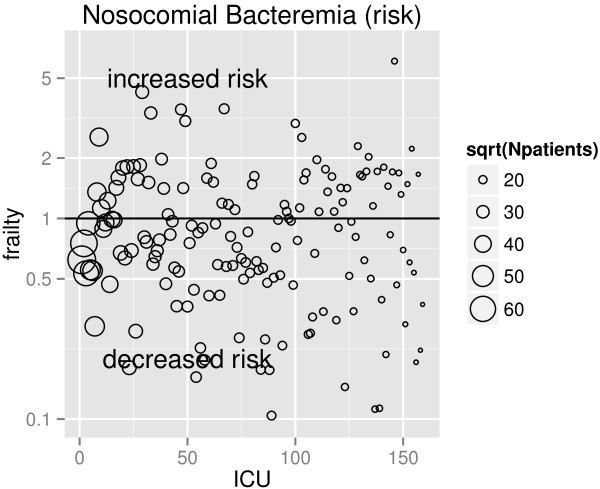
**Estimated frailties (random effects) for each ICU.** Data are from the shared frailty model for the subdistribution hazard of NB without covariates. The ICUs are ordered on the *X*-axis according to the contributing number of patients; the circles are proportional to the magnitude of contribution of the ICU, e.g., an ICU with sqrt(Npatients) = 60 contributed 60^2^ = 3600 admissions to the cohort. The *Y*-axis has a log scale.

The corresponding ICU effects (random effects or frailties) of the null models are shown in Figure 3; for instance, an ICU effect of 2 means that the baseline hazard of this ICU is twice as large as the hazard averaged over all ICUs. The observed heterogeneity across ICUs in the rates of NB is remarkably large (*θ* = 0.26 with standard error (SE) = 0.038), in contrast to the heterogeneity in rates of death without NB (*θ* = 0.14 with SE = 0.019) and discharge without NB (*θ* = 0.15 with SE = 0.017). The heterogeneity in risks of NB is even larger than in the rates (estimated by *θ* = 0.64 with SE = 0.076); see Figure 4. Large heterogeneity means that event times are strongly correlated within ICUs. One might conclude that observed and unobserved ICU-specific factors play a more substantial role in the NB hazard rates compared to the competing event rates.

Including covariates in the models led to only a small reduction in ICU-level heterogeneities for all three of the competing outcomes, with *θ* falling to 0.19 for NB, still larger than the heterogeneities in rates of the competing events (Table 2). Separate models (only ICU-level factors and only patient-level factors) showed that ICU-level factors reduced heterogeneity more than the patient-level factors (Table 2). It follows that the impact of unobserved ICU-specific factors is large. The heterogeneity reduction was stronger in the NB risk than the NB rate model. There are patient-level factors, such as the APACHE II score, type of diagnosis and trauma (Table 2), that have a strong effect in reducing the discharge without NB hazard (the strongest competing risk hazard), i.e., patients with high APACHE II scores or trauma stay longer in ICU. There are also ICU-level factors that are more pronounced: number of beds in ICU and teaching hospital. Thus, there is an indirect effect on the risk for NB leading to increased subdistribution hazard ratios. And strong effects lead to a reduction in heterogeneity.

**Table 2:** Results from multivariate analysis

**Risk factors**	**Event-specific analysis**			**Subdistribution analysis**
	**Nosocomial bacteremia (NB)**	**Death without NB in ICU**	**Discharge without NB**	**Nosocomial bacteremia**
	HR (95% CI)	HR (95% CI)	HR (95% CI)	Subdistribution HR (95% CI)
**ICU / hospital-level covariates**				
Number of beds in ICU:				
11–20 vs 0–10	1.18 (0.96–1.46)	1.01 (0.87–1.17)	1.43 (1.23–1.67)	1.34 (0.99–1.81)
21–30 vs 0–10	1.31 (0.97–1.77)	0.86 (0.69–1.08)	1.15 (0.89–1.47)	2.17 (1.42–3.30)
31–40 vs 0–10	1.56 (0.98–2.50)	0.81 (0.57–1.14)	1.83 (1.22–2.74)	1.85 (0.95–3.62)
> 40 vs 0–10	1.09 (0.55–2.18)	1.00 (0.60–1.65)	1.37 (0.76–2.48)	1.37 (0.50–3.72)
Number of beds in hospital:				
> 1000 vs 0–500	1.30 (0.85–1.99)	0.94 (0.70–1.27)	1.30 (0.90–1.87)	1.32 (0.71–2.47)
501–1000 vs 0–500	1.11 (0.91–1.36)	1.04 (0.90–1.21)	1.13 (0.95–1.34)	1.16 (0.87–1.53)
Teaching hospital (only) vs university+teaching	1.00 (0.82–1.22)	1.03 (0.89–1.20)	1.87 (1.61–2.18)	0.94 (0.71–1.26)
No teaching hospital vs university+teaching	0.71 (0.51–0.97)	1.08 (0.86–1.34)	2.42 (1.92–3.03)	0.59 (0.38–0.91)
Type of hospital (private vs public)	0.98 (0.70–1.39)	0.82 (0.64–1.05)	0.80 (0.61–1.05)	1.18 (0.76–1.85)
**Type of ICU:**				
Medical vs polyvalent	0.71 (0.44–1.14)	1.07 (0.78–1.47)	1.44 (0.99–2.10)	0.57 (0.30–1.08)
Surgery vs polyvalent	1.07 (0.62–1.85)	0.60 (0.40–0.91)	1.20 (0.73–1.96)	1.13 (0.51–2.50)
Coronary vs polyvalent	0.71 (0.28–1.82)	1.27 (0.64–2.51)	0.83 (0.44–1.57)	0.73 (0.24–2.28)
Traumatology vs polyvalent	1.20 (0.79–1.82)	1.03 (0.76–1.40)	1.28 (0.90–1.81)	1.15 (0.64–2.05)
Post-surgery cardiology vs polyvalent	1.47 (0.85–2.54)	0.71 (0.46–1.08)	1.95 (1.27–3.01)	1.39 (0.63–3.05)
Burn vs polyvalent	0.48 (0.16–1.42)	0.70 (0.31–1.62)	0.70 (0.31–1.59)	1.20 (0.30–4.79)
**Calendar year of admission**				
2007 vs 2006	1.02 (0.92–1.13)	1.01 (0.94–1.08)	0.95 (0.92–0.97)	1.02 (0.93–1.13)
2008 vs 2006	0.98 (0.89–1.08)	1.09 (1.02–1.16)	0.93 (0.91–0.95)	1.03 (0.93–1.14)
2009 vs 2006	1.09 (0.99–1.19)	1.02 (0.96–1.09)	0.86 (0.83–0.88)	1.19 (1.09–1.31)
2010+ vs 2006	0.84 (0.76–0.93)	1.08 (1.01–1.15)	0.94 (0.91–0.96)	0.83 (0.75–0.91)
**Patient level covariates**				
APACHE II score:				
11–20 vs 0–10	1.30 (1.20–1.42)	1.99 (1.85–2.14)	0.53 (0.52–0.54)	2.81 (2.59–3.06)
21–30 vs 0–10	1.38 (1.26–1.50)	4.11 (3.83–4.41)	0.28 (0.27–0.28)	4.54 (4.15–4.96)
> 31 vs 0–10	1.54 (1.37–1.73)	6.65 (6.15–7.19)	0.17 (0.17–0.18)	5.78 (5.14–6.49)
Age (years):				
0–40 vs 61–80	1.08 (0.99–1.18)	0.59 (0.55–0.64)	1.05 (1.03–1.07)	1.12 (1.02–1.22)
40–60 vs 61–80	1.09 (1.02–1.16)	0.79 (0.76–0.83)	0.98 (0.97–1.00)	1.18 (1.11–1.26)
> 80 vs 61–80	0.77 (0.68–0.86)	1.65 (1.57–1.73)	1.18 (1.15–1.21)	0.51 (0.45–0.57)
**Days in hospital before ICU admission:**				
4–6 vs 0–3	1.12 (1.00–1.25)	1.09 (1.01–1.17)	0.86 (0.83–0.89)	1.27 (1.13–1.42)
6–10 vs 0–3	1.14 (1.01–1.28)	1.18 (1.10–1.27)	0.85 (0.82–0.87)	1.33 (1.18–1.49)
> 10 vs 0–3	1.17 (1.08–1.27)	1.22 (1.16–1.29)	0.78 (0.76–0.80)	1.43 (1.32–1.55)
**Type of diagnosis:**				
Respiratory vs cardiovascular	0.86 (0.80–0.94)	0.93 (0.88–0.98)	0.63 (0.62–0.65)	1.43 (1.32–1.55)
Gastrointestinal vs cardiovascular	1.18 (1.09–1.29)	0.96 (0.91–1.02)	0.78 (0.76–0.79)	1.65 (1.51–1.80)
Central nervous system vs cardiovascular	0.91 (0.84–0.99)	1.41 (1.34–1.49)	0.65 (0.64–0.66)	1.38 (1.28–1.50)
Other diagnoses vs cardiovascular	1.20 (1.08–1.32)	0.78 (0.71–0.85)	0.76 (0.74–0.78)	1.96 (1.78–2.16)

Antibiotic treatment 48 h before and/or after ICU admission	0.83 (0.77–0.88)	1.08 (1.04–1.13)	0.70 (0.69–0.71)	1.01 (0.95–1.09)
Gender	1.08 (1.01–1.14)	1.01 (0.97–1.05)	0.99 (0.98–1.01)	1.12 (1.06–1.18)
Origin (hospital/ICU vs community)	1.05 (0.98–1.12)	0.98 (0.94–1.02)	1.00 (0.99–1.02)	1.09 (1.02–1.17)
Trauma	1.13 (1.04–1.24)	0.67 (0.62–0.72)	0.64 (0.62–0.66)	1.81 (1.66–1.98)

Variance of heterogeneity (without covariates)	0.26 (SE 0.038)	0.14 (SE 0.019)	0.15 (SE 0.017)	0.64 (SE 0.076)
Variance of heterogeneity (with ICU-level covariates only)	0.20 (SE 0.030)	0.12 (SE 0.015)	0.09 (SE 0.011)	0.47 (SE 0.061)
Variance of heterogeneity (with patient-level covariates only)	0.25 (SE 0.037)	0.12 (SE 0.016)	0.17 (SE 0.025)	0.56 (SE 0.069)
Variance of heterogeneity (with all covariates)	0.19 (SE 0.030)	0.11 (SE 0.015)	0.17 (SE 0.024)	0.40 (SE 0.052)

CI, confidence interval; HR, hazard rate; NB, nosocomial bacteremia; SE, standard error.

### Multilevel risk factors: patient level

Results from the risk factor analysis on the patient as well as ICU level are shown in Table 2. The hazard ratios for NB from the event-specific analysis (model 1) reflect direct effects on the occurrence of NB; this event-specific approach also shows how these factors are associated with the competing events, i.e., those which have potentially indirect effects on NB. The subdistribution analysis (model 2) is a summary analysis and studies the effects on the risk of NB (as a cumulative incidence function). Factors, i.e., an exposure, with a hazard ratio lower than 1 for the competing events increase the risk of NB since exposed patients stay longer in the ICU leading to more NBs in the exposed group. For instance, traumatic patients are associated with an increased *daily* risk of NB (hazard rate = 1.13) and also with a decreased *daily* risk of being discharged or dying without NB (both hazard rates lower than 1). In other words: traumatic patients acquire NB more frequently *per day* and in addition, as they stay longer at ICU, their extended time at ICU is itself a risk factor for NB, which causes them to acquire more NBs eventually. This indirectly adds to the cumulative risk of NB (subdistribution hazard rate = 1.81).

In a similar way, factors with a hazard ratio greater than 1 for the competing events decrease the risk of NB. For example, patients older than 80 years acquire fewer NBs *per day* than patients aged 61 to 80 years (hazard rate = 0.77) but they also die (and are discharged) more frequently *per day* at ICU (both hazard rates are greater than 1). This indirect effect additionally reduces the cumulative risk of NB (subdistribution hazard rate = 0.51). Often, the hazard ratios for discharge and death without NB are diametrically opposed; then, the discharge hazard has usually a stronger indirect effect because of its larger magnitude compared with the death hazard (Figure 1). For instance, the APACHE II score is highly associated with an increased rate of NB but also highly associated with an increased death (without NB) rate and a decreased discharge (without NB) rate (i.e., with a longer ICU stay). The indirect effect due to an extended length of ICU stay makes patients with higher APACHE II score acquire an NB more frequently, which is quantified by the subdistribution hazard ratios. It is even possible for a hazard ratio for NB to be lower than 1 but for the subdistribution hazard ratio to be larger than 1 (such as respiratory vs cardiovascular diagnosis). This is due to an indirect effect on the competing events: patients with a respiratory diagnosis stay much longer in ICU without NB than patients with a cardiovascular diagnosis, or in other words, their daily risk of being discharged or dying without NB is reduced (hazard ratios of both competing events are lower than 1). This additional impact on the risk of NB is so strong that respiratory patients acquire more NB than cardiovascular patients even though their daily risk of NB is reduced. These results highlight just some the complexities of examining risk factors of NI in the presence of competing risks.

### Multilevel risk factors: ICU level

A larger number of beds in an ICU was somewhat indirectly associated with an increased risk of NB (Table 2) because the number of beds in ICUs were moderately associated with the competing events. The rate of NB was higher in university/teaching hospitals compared with those without teaching, an effect which has also been found elsewhere [18]. This effect is even more pronounced in the subdistribution model because patients in non-teaching hospitals are discharged quicker.

## Discussion

In this paper, we used two multilevel competing risks models to evaluate risk factors of NI. To our knowledge, this is the first study to investigate the heterogeneity across ICUs in risks and rates of NI using a large multicenter cohort accounting for both ICU clustering effects and competing risks. Further, we showed that it is necessary to perform a multilevel competing risk analysis to understand fully the direct and indirect effects of risk factors on the occurrence of NI.

Our findings have the following implications. First, the large heterogeneity indicates that the impact of unobserved ICU-specific factors on the risk and rate of NI is large, even after accounting for important patient- and observed ICU-level characteristics. Thus, surveillance networks are encouraged to collect further potential ICU-level risk factors in addition to patient-level data. This large heterogeneity might also explain why ICU-based infection control strategies might work in some ICUs but not in others. It emphasizes the need for multicenter intervention studies rather than single-center pilot studies [19,20]. Ignoring heterogeneity in the analysis of multicenter studies can lead to biased results and misleading conclusions. In our cohort, the risks of NI were more heterogeneous than rates of NI across ICUs. From the mathematical point of view, this is not necessarily the case since different correlations between competing hazard constellations could potentially result in a very similar risk of NI.

Second, competing risks play an essential role in the understanding of NI occurrence and the analysis must account for this [7]. The distinction between indirect and direct effects is a key issue for understanding the associations between risk factors and NI outcomes. For instance, a cohort study of African children reported no association between burns and pediatric hospital-acquired bacteremia [21] even though the cumulative risk is about three times higher, due to competing events [7]. Therefore, we recommend that the results from both models (the rate and the risk models) are reported [22] to make hidden and indirect effects transparent. This is very important for NI since risk factors for NI are often also associated with the competing risks for NI as well. This may result in more pronounced effects in the risk model (such as APACHE II score, type of diagnosis or trauma) or effects that are relevant only in the rate model but irrelevant in the risk model (e.g., antibiotic treatment before ICU admission). It could even lead to apparent diametrically opposed results (e.g., both respiratory and central nervous system vs cardiovascular diagnoses). Third, our approach has the potential to identify ICUs with unusually high or low rates. Nevertheless, we emphasize that extreme caution is needed when using NI rates or risks for benchmarking or as quality indicators. Besides problems in reliability, validity [23] and statistical uncertainty, there are further complications from competing risks as shown here.

There are some limitations to our study. As in other large surveillance studies based on volunteer ICUs, data can be subject to reporting, information or selection bias. Therefore, it is possible that part of the heterogeneity might be attributed to over- or under-reporting of NI rather than to real ICU factors [24]. Information bias might occur due to unreported NB cases. As suggested by Hansen *et al*. [18], surveillance data need to be validated to counter differences across ICUs. Further, an extrapolation of our findings to other European countries is limited due to differences in ICU management.

Multilevel analyses in hospital infection epidemiology are still rare but necessary to evaluate effects of individual-level and group-level factors [4]. Vellinga *et al*. have produced a valuable overview of the principles of multilevel analysis for antimicrobial resistance studies [5]. We have extended their methodology to complex survival data for hospital infections because modeling the timing of events (infection, death or discharge) is crucial.

## Conclusions

We encourage further investigations using our methodological approaches, e.g., to evaluate the occurrence of antimicrobial resistance by exploring antibiotic usage at the patient level and ICU level simultaneously. Statistical models and corresponding codes are available in Additional file 1.

## Key messages

• Discharge from and death in an intensive care unit are competing risks for nosocomial infection.

• There are factors on the patient as well as on the ICU level influencing the occurrence of nosocomial infections.

• Analysis of data from large multicenter studies has the potential to improve our understanding of how patient and ICU-level characteristics impact nosocomial infections.

• A combination of multilevel and competing risk models are necessary to analyze such complex data.

• We encourage further investigations by using our methodological approaches to evaluate our findings of unexplained heterogeneity. The statistical code is available in Additional file 1.

## Abbreviations

CI: confidence interval; HR: hazard rate; ICU: intensive care unit; NB: nosocomial bacteremia; NI: nosocomial infection; SE: standard error.

## Competing interests

The authors declare that they have no competing interests.

## Authors’ contributions

MW built the model, performed the statistical analysis and drafted the manuscript. BSC, AGB, SH and MS were involved in model building and helped to draft the manuscript. MPM, FAL and POA participated in the design and coordination of the HELICS-ENVIN study and drafted the manuscript for clinical aspects. All authors read, revised and approved the final manuscript.

## Supplementary Material

Additional file 1 Appendix: Statistical methods and R code for multilevel competing risk models. Click here for file
