# Supplementary material for: Multilevel competing risk models to evaluate the risk of nosocomial infection
Source: Crit Care. 2014 Apr 8;18(2):R64. doi: 10.1186/cc13821 (PMC4056071; doi:10.1186/cc13821)

Appendix:
Multilevel competing risk models to evaluate the risk of nosocomial infection
# - Statistical methods and R code -

December 9, 2013

Statistical methods

Model 1: Shared frailty models for event-specific hazard rates - the etiology (rate) model

The classical way to analyse competing risks data is to study event-specific hazard rates. We introduce random effects for each ICU by a shared gamma frailty model [1, 2]. Thus, for each event k (NI, death w/o NI, discharge w/o NI), for the j-th patient in the i-th ICU, the event-specific hazard rate with a shared frailty term Z_i^k is defined as

$$\lambda_{ij}^k(t|Z_i^k) = Z_i^k \lambda_0^k(t) \tag{1}$$

The frailty term Z_i^k is a random effect which varies across ICUs (patients within ICU share the same frailty) and is assumed to be Gamma distributed with shape parameter $1/\theta_k$ and inverse scale parameter $1/\theta_k$, thus $E(Z^k) = 1$ and $Var(Z^k) = \theta_k$. Large values of θ_k signify a closer positive relationship between patients within ICU and greater heterogeneity across ICUs. The baseline hazard for event k is labeled as $\lambda_0^k(t)$.

Observed covariates X are considered in a multiplicative way as follows

$$\lambda_{ij}^k(t|X_{ij}, Z_i^k) = Z_i^k \lambda_0^k(t) exp(\beta^k X_{ij}) \tag{2}$$

We consider patient-individual as well as ICU-specific covariates.

Model 2: Shared frailty model for the subdistribution hazard of NI - the prediction (risk) model

For studying the risk (cumulative incidence function) of NI, we use the Fine and Gray model [3] and introduce a shared frailty structure to investigate heterogeneity in a similar way as Katsahian et al [4]. Thus, we set all event times to time until potential censoring for patients who are discharged and died w/o NI [5]. These times are known because of administrative censoring. Then we fit shared frailty models in analogy to formula 1 and 2 to this modified data set.

Table 1: Hypothetical data. ICU ID=identity code for the ICU, patient ID = identity code for the patient, time=time from admission, to=type of event (1=NI, 2=death without NI, 3=discharge without NI, 0=censored), timepotcens=time from admission to potential censoring, timesubNI=subdistribution time for NI (times of competing events are replaced with timepotcens), left-truncated data should use the start-stop notation

ICU ID	patient ID	time	to	timepotcens	timesubNI	covariates
1	1	5	1	45	5	0
1	2	8	3	65	65	1
1	3	4	2	73	73	1
1	4	17	1	123	17	1
1	5	9	3	47	47	0
1	6	5	3	79	79	0
2	7	7	3	76	76	1
2	8	8	3	65	65	1
2	9	14	1	123	14	1
2	10	12	2	54	54	0
2	11	9	1	76	9	0
2	12	9	0	89	9	1
(...)	(...)	(...)	(...)	(...)	(...)	(...)

Data analysis

Software

For all analyses we used the flexible R-package *frailtyPack* [6].

R code

```
# load the software
library("frailtypack")

# data should look like in table 1
# note that data$timesubNI<-ifelse(data$status==1,data$time,data$timepotcens)

# run model 1 and formula 1 for nosocomial infection using
# semi-parametric hazard functions with the penalized likelihood estimation
model1_NI<-frailtyPenal(Surv(time,to==1) ~ cluster(ICU_id) ,
           data=data,n.knots=7,kappa1=1000,Frailty=TRUE,cross.validation=TRUE)

# plot baseline hazard
plot(model1_NI)

# plot frailties
plot(model1_NI$frailty.pred)

# print results
```

```
print(model1_NI)
summary(model1_NI)

# perform the same analysis for death without NI by
# replacing Surv(time,to==1) by Surv(time,to==2)

# perform the same analysis for discharge without NI by
# replacing Surv(time,to==1) by Surv(time,to==3)

# Multivariate model
# run model 1 and formula 2 for nosocomial infection using
# semi-parametric hazard functions with the penalized likelihood estimation
model1_NI_multi<-frailtyPenal(Surv(time,to==1) ~ cluster(ICU_id) + covariates ,
           data=data,n.knots=7,kappa1=1000,Frailty=TRUE,cross.validation=TRUE)

# Subdistribution model
# run model 2 for nosocomial infection using
# semi-parametric hazard functions with the penalized likelihood estimation
model2_NI<-frailtyPenal(Surv(timesubNI,to==1) ~ cluster(ICU_id)  ,
           data=data,n.knots=7,kappa1=1000,Frailty=TRUE,cross.validation=TRUE)


model2_NI_multi<-frailtyPenal(Surv(timesubNI,to==1) ~ cluster(ICU_id) + covariates,
           data=data,n.knots=7,kappa1=1000,Frailty=TRUE,cross.validation=TRUE)
```

References

[1] Liquet B, Timsit JF, Rondeau V. Investigating hospital heterogeneity with a multi-state frailty model: application to nosocomial pneumonia disease in intensive care units. BMC Med Res Methodol. 2012;12:79.

[2] Rondeau V, Commenges D, Joly P. Maximum penalized likelihood estimation in a gamma-frailty model. Lifetime Data Anal. 2003 Jun;9(2):139–153.

[3] Fine J, Gray RJ. A proportional hazards model for the subdistribution of a competing risk. J Am Stat Assoc. 1999;94(446):496–509.

[4] Katsahian S, Boudreau C. Estimating and testing for center effects in competing risks. Stat Med. 2011 Jun;30(13):1608–1617.

[5] Beyersmann J, Allignol A, Schumacher M. Competing Risks and Multistate Models with R. Springer, New York; 2011.

[6] Rondeau V, Mazroui Y, Gonzalez JR. frailtypack: An R package for the analysis of correlated survival data with frailty models using penalized likelihood estimation or parametrical estimation. Journal of Statistical Software. 2012;47(4):1–28.

Additional information: boxplots of the frailties as an alternative display of the data published in the main manuscript

Figure 1: Outcome: NB. model 0 - null model, model 1 - with ICU level covariates only, model 2 - with patient level covariates only, model 3 - all covariates

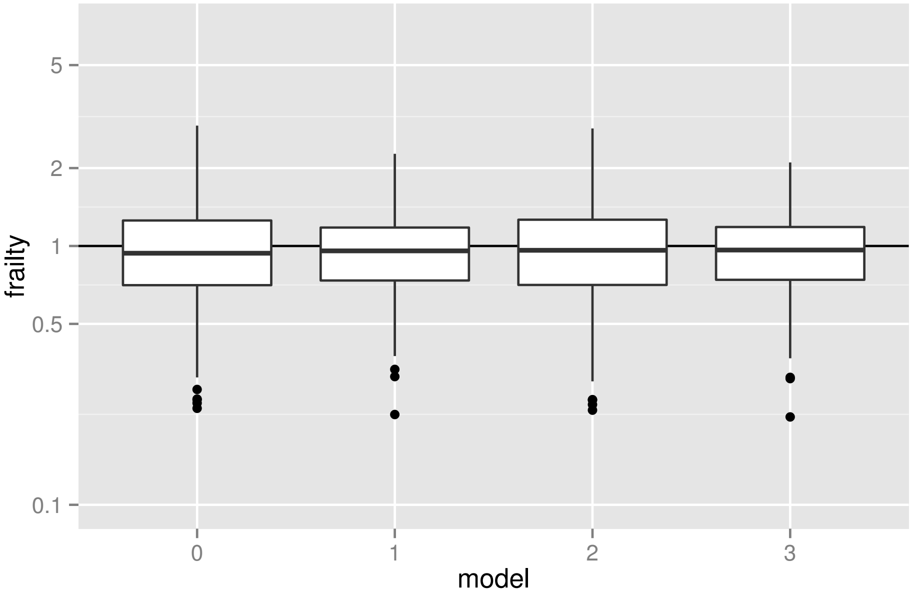

Figure 2: Outcome: death without NB. model 0 - null model, model 1 - with ICU level covariates only, model 2 - with patient level covariates only, model 3 - all covariates

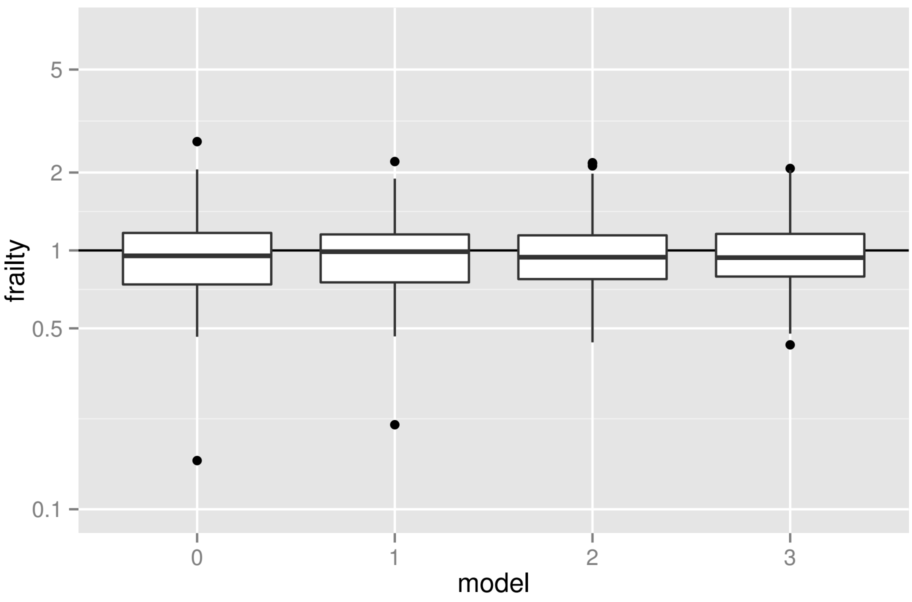

Figure 3: Outcome: discharge without NB. model 0 - null model, model 1 - with ICU level covariates only, model 2 - with patient level covariates only, model 3 - all covariates

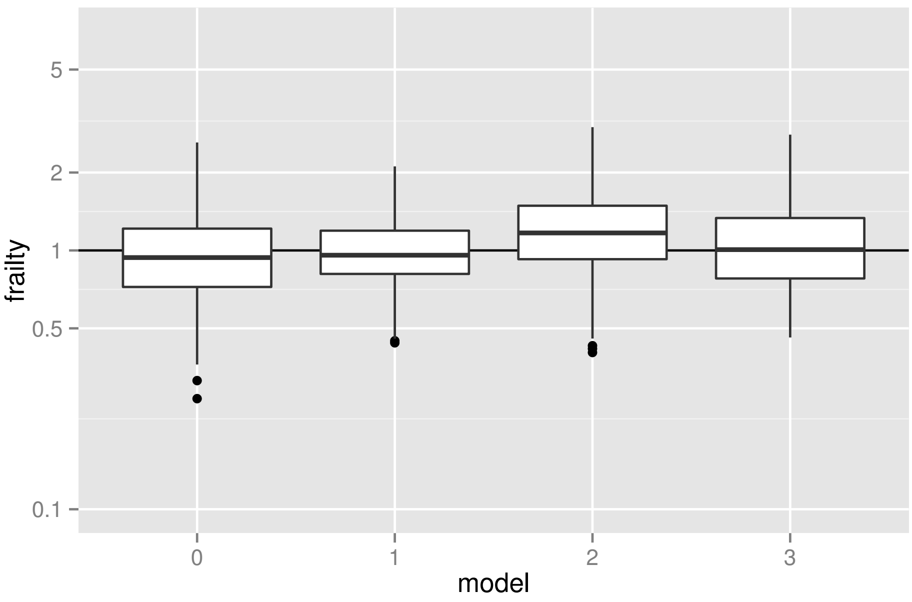

Figure 4: Outcome: NB subdistribution. model 0 - null model, model 1 - with ICU level covariates only, model 2 - with patient level covariates only, model 3 - all covariates

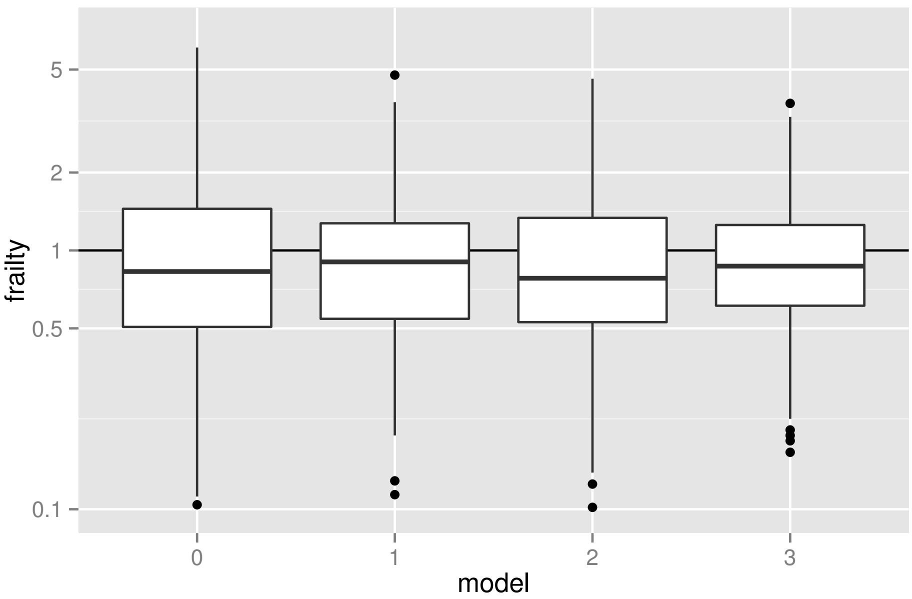

Supplement: Additional file 1 — Appendix: Statistical methods and R code for multilevel competing risk models. [file cc13821-S1.pdf]
